# MITA/STING and Its Alternative Splicing Isoform MRP Restrict Hepatitis B Virus Replication

**DOI:** 10.1371/journal.pone.0169701

**Published:** 2017-01-05

**Authors:** Shuhui Liu, Kaitao Zhao, Xi Su, Lu Lu, He Zhao, Xianwen Zhang, Yun Wang, Chunchen Wu, Jizheng Chen, Yuan Zhou, Xue Hu, Yanyi Wang, Mengji Lu, Xinwen Chen, Rongjuan Pei

**Affiliations:** 1 State Key Laboratory of Virology, Wuhan Institute of Virology, Chinese Academy of Sciences, Wuhan, China; 2 University of Chinese Academy of Sciences, Beijing, China; 3 Department of Microbiology, Tongji Medical College, Huazhong University of Science and Technology, Wuhan, China; 4 Institute of Virology, University Hospital of Essen, University of Duisburg-Essen, Essen, Germany; Indiana University, UNITED STATES

## Abstract

An efficient clearance of hepatitis B virus (HBV) requires the coordinated work of both the innate and adaptive immune responses. MITA/STING, an adapter protein of the innate immune signaling pathways, plays a key role in regulating innate and adaptive immune responses to DNA virus infection. Previously, we identified an alternatively spliced isoform of MITA/STING, called MITA-related protein (MRP), and found that MRP could specifically block MITA-mediated interferon (IFN) induction while retaining the ability to activate NF-κB. Here, we asked whether MITA/STING and MRP were able to control the HBV replication. Both MITA/STING and MRP significantly inhibited HBV replication *in vitro*. MITA overexpression stimulated IRF3-IFN pathway; while MRP overexpression activated NF-κB pathway, suggesting these two isoforms may inhibit HBV replication through different ways. Using a hydrodynamic injection (HI) mouse model, we found that HBV replication was reduced following MITA/STING and MRP expression vectors in mice and was enhanced by the knockout of MITA/STING (MITA/STING^-/-^). The HBV specific humoral and CD8^+^ T cell responses were impaired in MITA/STING deficient mice, suggesting the participation of MITA/STING in the initiation of host adaptive immune responses. In summary, our data suggest that MITA/STING and MRP contribute to HBV control via modulation of the innate and adaptive responses.

## Introduction

Hepatitis B virus (HBV) is a noncytopathic, hepatotropic double-stranded DNA virus that causes acute and chronic hepatitis and increases the risk of hepatic cirrhosis and hepatocellular carcinoma (HCC). It is generally accepted that the vigor and quality of the cellular immune response determined whether HBV infection is cleared or persists. The adaptive immune responses to viral antigens, especially the destruction of infected cells and the non-lytic mechanisms eliminating HBV cccDNA mediated by CD8 cytotoxic T lymphocytes (CTLs), are responsible for HBV clearance in HBV acute infection [1–3].

In addition to the adaptive immune response, the innate immune response induced by the recognition of pathogen-associated molecular patterns (PAMPs) via pattern recognition receptors (PRRs) plays an indispensable role in controlling viral infection [4]. Many PRRs have been identified to be nucleic acid sensors, such as toll-like receptors (TLRs) [5], nucleotide-binding oligomerization domain leucine-rich repeat proteins (NOD-like receptors, NLRs) [6], retinoic acid-inducible gene I (RIG-I)-like receptors (RLRs) [7], the member of the PYHIN protein family Interferon Gamma Inducible Protein 16 (IFI16) and proteins absent in melanoma 2 (AIM2) [8–11], DNA-dependent activator of IFN regulatory factors (DAI) [12], the member of the DEXDc family of helicases DDX41 and cyclic GMP-AMP (cGAMP) synthase (cGAS) [13–15]. Once the PRR senses PAMPs, a series of signaling cascades are triggered, resulting in the activation of transcription factors IRF3 and NF-κB, then the following production of interferon (IFN) and inflammatory cytokines, which restrict microbial invasion and further evocate the adaptive immune responses [16].

With regard to HBV, it was thought to be a stealthy virus because the innate responses are predominantly weak or absent during the natural course of HBV infection [17, 18]. However, it is possible that HBV is detected by the innate immune system, while the virus can actively suppress or evade the early antiviral response. Indeed, recent studies have shown that the pgRNA (pregenomic RNA) of HBV and the cytoplasmic exposed HBV rcDNA (relaxed circular DNA), a result of nucleocapsid destabilization, could be detected by RIG-I and MITA/STING, respectively [19, 20]. The activation of different innate immune signaling pathways may induce type III IFN response and inhibit HBV replication [20]. On the other hand, accumulating evidences have demonstrated that distinct HBV proteins and HBV virion particles could impair the innate immune signaling such as RIG-I, TLR3 and STING/MITA-stimulated signaling through various mechanisms. HBV-induced miR146a could target RIG-I thus attenuate IFN production [21]. The HBV X protein interacts with MAVS and the P protein competes for DDX3 binding with TBK1, to inhibit RIG-I-mediated type I IFN pathway signaling [22, 23]. HBV P protein blocks STING/MITA mediated innate immunity response by disrupting K63-linked ubiquitination of STING/MITA [24].

MITA/STING is an adapter protein in the cellular signaling cascade downstream of the DNA sensor cGAS and RNA sensor RIG-I [16]. As reported previously, the cGAS-STING signaling pathway could recognize several invading viruses such as herpes simplex virus type 1 (HSV-1), vaccinia virus (VACV), and human immunodeficiency virus (HIV) [14, 25–27]. In addition, MITA/STING also plays an important role in IFNs production during Dengue virus (DENV) infection [28]. An alternatively spliced variance of MITA/STING, designated as MITA-related protein (MRP), was found to act as a dominant negative mutant of MITA/STING, blocking MITA/STING-mediated IFN induction by disrupting the interaction between MITA/STING and TBK1; interestingly, MRP is still able to activate NF-κB [29]. Because dsDNA and ssDNA are produced during HBV replication, we propose that MITA and MRP are involved in the recognition of HBV replicative intermediates and the regulation of HBV clearance. A recent study demonstrated that activation of cGAS-STING pathway could contribute to the control of HBV replication [30]. Here, we compared the effect of MITA/STING and MRP on cellular signaling as well as their effect on HBV replication in Huh7, HepG2.2.15 cells and in mice. We found that MRP, despite its inability to trigger IRF3 activation, could restrict HBV replication, suggesting the importance of NF-κB-mediated antiviral functions. Interestingly, MITA/STING deficiency resulted in enhanced HBV replication and lower specific humoral and CD8+ T cell immune responses to HBV in the hydrodynamic injection mouse model, suggesting that MITA/STING plays an important role in triggering HBV specific adaptive immune responses.

## Materials and Methods

### Ethics statement

This study was performed in strict accordance with recommendations in the Guide for the Care and Use of Laboratory Animals according to the regulation in the People’s Republic of China. MITA/STING^-/-^ mice, kindly provided by Prof. H. B. Shu (Wuhan University, Wuhan) [31], and littermate control wild-type (WT) mice were bred and maintained under specific pathogen-free conditions at the Central Animal Laboratory of Wuhan Institute of Virology, Chinese Academy of Sciences (WIV, CAS. License number: SYXK2014-0034). All animal experiments were approved by the Institutional Animal Ethical Committee of WIV, CAS (Serial number: WIVA02201404). All procedures were carried out under isoflurane anesthesia and all mice were monitored, weighted daily. None of the mice became severely ill or died prior to the experimental endpoint. The mice were anesthetized with isoflurane and then euthanized by cervical dislocation at the experimental endpoint. All efforts were made to minimize any suffering and the number of animals used in the study.

The knockout mice were screened by genotyping. The mice genomic DNA was extracted from digested tail tissue and genotyping was performed by PCR with the following primers. Knockout and wild type mice shared common forward primer: 5’-CTCCTAGACAGGTGCTGTAGGATG-3’. Reverse primer for knockout mice, 5’-AAGGGTTATTGAATATGATCGGA-3’; reverse primer for wild type, 5’-TGGAGACCACAGAGGGTTACCTG-3’ (data not shown). The bone marrow cells were collected from mice long bones and the progenitor cells were differentiated into bone marrow derived macrophage (BMDM) with L929 cell condition medium as previously described [32]. The knockout of MITA/STING was further confirmed by enhanced susceptibility of BMDM to vesicular stomatitis virus (VSV) (data not shown).

### Plasmids, reagent and cell culture

The HBV replication competent plasmid pSM2 was kindly provided by Prof. Dr. Hans Will (Heinrich-Pette-Institute, Hamburg, Germany) [33]. The plasmid was constructed by cloning the *Eco*RI head-to-tail dimer of HBV genome (genotype D), subtype *ayw* (GenBank accession number, V01460) into plasmid pMa5-8 [33, 34], using the authentic HBV promoter for HBV transcription. The human MITA/STING overexpression plasmid pFlag-MITA (pMITA), human MRP overexpression plasmid pHA-MRP (pMRP) and control vector plasmid pcDNA 3.1(+) were described previously [29]. The reporter plasmids pIFN-β-luc, pIRF3-luc, pNF-κB-luc, pISRE-luc were purchased from Clontech. pRL-TK was purchased from Promega. The human β-actin expression plasmid pHA-β-actin was generated by cloning the β-actin sequence into pXJ-40 with *Xho*I and *Kpn*I and used as a control for assessing the transfection efficiency. The MITA/STING ligand c-di-GMP was purchased from InvivoGen. The NF-κB inhibitor ammonium pyrrolidinedithiocarbamic (PDTC) was purchased from Beyotime (Shanghai, China). The human hepatoma cell lines Huh7 and HepG2 were cultured in Dulbecco’s modified Eagle’s medium (DMEM) (Invitrogen) supplemented with 10% fetal bovine serum (FBS) (Invitrogen), 100 U/ml penicillin (Invitrogen), 100 mg/ml streptomycin sulfate (Invitrogen), 2 mM L-glutamine (Invitrogen) and nonessential amino acids (Invitrogen). HepG2.2.15 cells with integrated dimers of the HBV genome (GenBank accession number, U95551) was cultured with 500 μg/ml of G418 (Sigma-Aldrich).

### Transfection and luciferase assays

Transient transfections of Huh7, HepG2 and HepG2.2.15 cells were performed with Lipofectamine 2000 (Invitrogen) according to the manufacturer’s instructions. Cells were transfected with the indicated amount of pFlag-MITA or pHA-MRP combined with pSM2. For the luciferase assays, cells were transfected with the indicated amount of pFlag-MITA or pHA-MRP and 100 ng of the indicated reporter plasmids. For the inner control, 10 ng of pRL-TK was co-transfected. The reporter activity was measured at 18–24 hours post-transfection (hpt) using the Dual-Luciferase reporter assay system (Promega).

### Enzyme-linked immunosorbent assay (ELISA) and Western blot analysis

To measure the levels of HBsAg and HBeAg, culture supernatants of transfected cells were diluted 5-fold and analyzed by ELISA as described previously [35]. Levels of HBsAg, HBeAg and antibody to HBsAg in mouse sera were measured by ELISA after a 1:10 dilution [35]. Western blot analysis was performed as previously described [35]. The following antibodies were used: rabbit polyclonal anti-HBc and mouse monoclonal antibodies (mAbs) against β-actin from Santa Cruz Biotechnology, anti-GAPDH (Proteintech), anti-FLAG-tag and rabbit monoclonal Abs against HA-tag from Sigma-Aldrich, anti-phospho-IRF3 (Ser396), anti-IRF3, anti-phospho-IκB (Ser32), anti-IκB, anti-phospho-NF-κB p65 (Ser536) and anti-NF-κB p65 from Cell Signaling Technology. Proteins were visualized with appropriate HRP-conjugated secondary antibodies (Jackson Immuno Research) and SuperSignal-Femto chemiluminescent substrate (Pierce).

### Detection of HBV DNA replicative intermediates and HBV core-associated DNA by Southern blot or real-time PCR

For extraction of HBV replicative intermediates, Huh7 or HepG2.2.15 cells were lysed in 800 μl of DNA extraction buffer (50 mM Tris-HCl, 50 mM NaCl, 1 mM EDTA, 1% NP-40, pH 7.4) with 10 mM MgCl_2_ and 100 μg/ml DNase I (Sigma-Aldrich, St. Louis, MO) and maintained at 37°C for 0.5 h. The DNase I digestion was stopped using 25 mM EDTA (pH 8.5). Then, the samples were further digested with 5 mg/ml proteinase K (Qiagen) and 1% SDS at 55°C for 2 h. The HBV replicative intermediates were extracted with a phenol-chloroform mixture (1:1 ratio) and subjected to Southern blot analysis or quantitative real-time PCR as described previously [36].

Serum HBV core-associated DNA was extracted using a QIAamp DNA Blood Mini Kit (Qiagen). HBV DNA was quantitatively detected by real-time PCR using the SYBR green real-time PCR master mix (Roche). The primers used for real-time PCR were as follows: Forward, 5’- ACCAATCGCCAGTCAGGAAG-3’; Reverse, 5’- ACCAGCAGGGAAATACAGGC-3’.

### RNA interference

The following siRNAs were used: siControl (Qiagen #1027281); siRNA to human MITA/STING (Qiagen #SI04287626) and siRNA synthesized specific to MRP (GenePharm^TM^, 5’-CGGGCAGCGGAACCUGCA-3’). The siMRP was designed and had been proved to be specific to MRP [29]. The siRNAs were transfected using Lipofectamine RNAiMax (Invitrogen) at a final concentration of 80 nM referring to the manufacturer’s instructions. To maintain the gene silencing effect from the beginning of virus replication until the last time point analyzed, the cells were split 24 hours after the initial transfection and then transfected with the same siRNA together with pSM2.

### HBV capsid detection

The procedure used to prepare intracellular capsid was adapted from the procedure by Roychouhury *et al*. [37]. Generally, the cultured cells were lysed in extraction buffer (10 mM Tris-HCl, 50 mM NaCl, 1 mM EDTA, 0.25% NP-40, 8% sucrose, pH 7.4) for 10 min on the ice. After centrifugation, the supernatant was collected and digested with 6 μM MgCl_2_, 0.2 mg/ml DNase I (Sigma-Aldrich, St. Louis, MO) and 1.5 mg/ml RNase (Omega) for 20 min at 37°C. After centrifugation, the supernatant was collected and subjected to electrophoresis on a 1.6% native agarose gel. Then the capsid was transferred to nitrocellulose filtermembrane and subjected to immunoblot analysis with rabbit polyclonal anti-HBV core antibody (Dako, GB058629).

### Immunoprecipitation of mature virions in supernatant

The supernatant of cells were collected and pre-incubated with 2 μg of monoclonal anti-HBs antibodies (A11, S11, and S1, kindly provided by Yan Bin, WIV, CAS, China) for overnight at 4°C [38]. Then the agarose beads protein A or protein G (Millipore) were added into mixture and rotated for 4 h at 4°C. After four times wash with phosphate buffered saline, the mixture was resuspended in 200 μl phosphate buffered saline and subjected to HBV core-associated DNA extraction and detection by real-time PCR [39].

### RNA extraction, quantitative real-time RT-PCR, and Northern blot analysis

Total RNA from cultured cells was extracted with TRIzol reagent (Invitrogen) and digested with RNase-free DNase (Promega). Specific mRNAs were quantified by one-step qRT-PCR using the QuantiTect SYBR Green RT-PCR kit (Qiagen). Primers used are listed in Table 1. The mRNA levels were normalized against the copy number of human beta-actin mRNA. Intracellular HBV mRNAs were detected using Northern blot analysis as described previously [40].

**Table 1:** Primers used for qRT-PCR.

primer	sequence
*Ifnb* Forward	5’-CACGACAGCTCTTTCCATGA-3’
*Ifnb* Reverse	5’-AGCCAGTGCTCGATGAATCT-3’
*Tnfa* Forward	5’-CACAGTGAAGTGCTGGCAAC-3’
*Tnfa* Reverse	5’-AGGAAGGCCTAAGGTCCACT-3’
*Il-6* Forward	5’-ACCCCCAATAAATATAGGACTGGA-3’
*Il-6* Reverse	5’-TTCTCTTTCGTTCCCGGTGG-3’
*Cxcl2* Forward	5’-CAAGAACATCCAAAGTGTGA-3’
*Cxcl2* Reverse	5’-CCATTCTTGAGTGTGGCTAT-3’
*Isg56* Forward	5’-GAAAGCCTCAGTCTTGCAGC-3’
*Isg56* Reverse	5’-CCTGTTGTAAGAGGCCAGCA-3’
*MxA* Forward	5’- CTCCGACACGAGTTCCACAA-3’
*MxA* Reverse	5’-GGCTCTTCCAGTGCCTTGAT-3’
*Cxcl10* Forward	5’-CCTGCAAGCCAATTTTGTCCA-3’
*Cxcl10* Reverse	5’-TGCATCGATTTTGCTCCCCT-3’
*Oas1* Forward	5’-ATTCTGCTGGCTGAAAGCAAC-3’
*Oas1* Reverse	5’-GGAGTGTGCTGGGTCTATGA-3’
*Beta-actin* Forward	5' TGGAATCCTGTGGCATCCATGAAAC 3'
*Beta-actin* Reverse	5' TAAAACGCAGCTCAGTAACAGTCCG 3'

### Mouse model of hydrodynamic injection

Hydrodynamic injection (HI) was performed as previously described [38]. In brief, 10 μg of HBV plasmid DNA was injected (5 to 8 s injection) alone or in combination with 10 μg of vector plasmid, pFlag-MITA or pHA-MRP into the tail veins of mice aged 6–8 weeks in a volume of phosphate buffered saline (PBS) equivalent to 8% of the body weight.

### Liver tissue preparation and immunohistochemistry (IHC) staining

Liver tissues were collected from mice sacrificed at 4 days post-hydrodynamic injection (days post-HI). To detect intrahepatic HBV replicative intermediates, 30 mg of mouse liver was lysed in 1 ml DNA extraction buffer, extracted as described above and subjected to Southern blot analysis.

To detect intrahepatic HBV RNAs, 50 mg of mouse liver was splintered and subjected to RNA extraction as described above. Of the purified RNA, 10 μg was used for Northern blot and another 10 μg was subjected to agarose gel electrophoresis as an RNA loading control (18S/28S). Relative band intensities of viral mRNAs were quantified using Image J software (NIH) [41].

For IHC staining, the collected liver samples were fixed in formalin and embedded in paraffin. Expression of pMRP, pMITA and intrahepatic HBcAg was detected by immunohistochemical staining with anti-HA, anti-Flag (Sigma-Aldrich) and anti-HBc antibodies (Dako, Carpinteria, CA), respectively, as described previously [42]. The samples were then probed with an appropriate horseradish peroxidase (HRP)-conjugated secondary antibody and visualized with the Envision system.

### Intrahepatic leukocytes and splenocytes isolation, intracellular cytokine staining and flow cytometry

To analyze T cell responses, mouse livers were perfused with phosphate buffered saline, homogenized, passed through a nylon mesh (BD, Biosciences, San Jose, CA), and then digested with 0.2 mg/ml collagenase-IV and 0.002% DNase I (approximately 50–100 U/ml) (Sigma-Aldrich, St. Louis, MO) for 30 min at 37°C. Hepatocytes were removed by centrifugation for 5 min at 60×g and the supernatant containing intrahepatic leukocytes (IHLs) was retained. The precipitant was washed with RPMI 1640 twice at 50×g for 5 min and collect the supernatant. The supernatant was then pelleted by centrifugation at 300×g for 10 min at 4°C and resuspended in RPMI 1640. The cell resuspension was gently layered on Mouse Lymphocyte Separation Medium (Dakewe, Beijing, China) and leukocytes were isolated according to the manufacturer’s protocols.

To isolate splenocytes, mouse spleens were homogenized and passed through a nylon mesh. The splenocytes were purified directly using Mouse Lymphocyte Separation Medium (Dakewe, Beijing, China) according to the manufacturer’s protocols.

Intracellular cytokine staining and flow cytometry analysis of intrahepatic lymphocytes and splenocytes were performed as previously described [35]. Peptides corresponding to the HBsAg CD8^+^ T cell epitope (K^b^/S_190–197_, VWLSVIWM) and the HBcAg CD8^+^ T cell epitope (K^b^/C_93–100_, MGLKFRQL) were used for lymphocyte stimulation. Allophycocyanin (APC)-conjugated anti-mouse IFN-γ, phycoerythrin (PE)-conjugated anti-mouse IL-2, fluorescein isothiocyanate (FITC)-conjugated anti-mouse TNF-α and APC-Cy7-conjugated anti-mouse CD8 mAbs (BioLegend, San Diego, CA) were used for flow cytometry. Dead cells were excluded by staining with 7-aminoactinomycin D (7-AAD; BioLegend, San Diego, CA). Flow cytometry data were acquired on FACS Caliburor or LSRII flow cytometers (BD, Biosciences). Data analysis was performed using FlowJo (Tree Star) software.

### Statistical analysis

Statistical analysis was performed using GraphPad Prism version 5 (GraphPad Software Inc., San Diego, CA). Significant differences were analyzed using a two-tailed unpaired *t*-test. The two ways ANOVA followed by Bonferroni’s test was used to determine the differences in multiple comparisons. P-values were calculated, and statistical significance is reported as highly significant using *(p<0.05), ** (p<0.01), or *** (p<0.001). Data are presented as the mean ± standard deviation.

## Results

### The activity of MITA/STING and MRP in Huh7 cells

**Fig 1:**
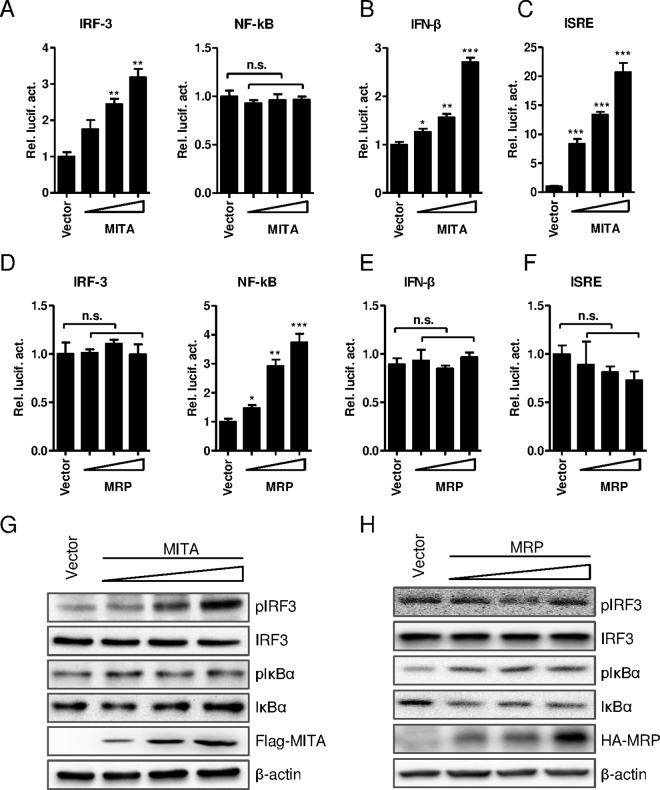
The functions of MITA/STING and MRP in hepatoma cell line Huh7 which supports HBV replication. (A) pIRF3-luc and pNF-κB-luc, (B) pIFN-β-luc and (C) pISRE-luc reporter plasmids were cotransfected with a serial concentration of pFlag-MITA/STING plasmids into Huh7 cells. (D) pIRF3-luc and pNF-κB-luc, (E) pIFN-β-luc and (F) pISRE-luc reporter plasmids were cotransfected with a serial density of pHA-MRP plasmids into Huh7 cells. The reporter activity was measured at 18–24 hpt with the Dual-Luciferase reporter assay system. (G-H) A serial increasing amount of pFlag-MITA or pHA-MRP plasmids were transfected into Huh7. The intracellular pIRF3, IRF3, pIκB and IκB protein levels were detected by Western blot. The two ways ANOVA followed by Bonferroni’s test was used to determine the differences in multiple comparisons (*, P <0.05; **, P <0.01; ***, P <0.001).

Although MRP was identified as a dominant negative mutant of MITA/STING, it retains the ability to activate NF-κB in 293T cells [29]. Therefore, it is worthwhile to investigate and compare the function of MITA/STING and MRP during HBV replication. The activities of MITA/STING and MRP in the IFN-β signaling pathway in Huh7 cells, a hepatoma cell line supporting HBV replication, were first analyzed using reporter assays. The results showed that MITA/STING overexpression activated IRF3- but not NF-κB-dependent luciferase expression in Huh7 cells (Fig 1A). The IFN-β promoter and the downstream ISRE-dependent reporter expressions were stimulated by MITA/STING in a dose-dependent manner (Fig 1B and 1C), but MRP overexpression only stimulated the NF-κB-dependent reporter and had no effect on IFN-β, IRF3 or the ISRE-dependent reporter expression (Fig 1D and 1F). Consistent with reporter results, the phosphorylation of IRF3 but not IκB was induced by MITA/STING in a dose-dependent way, while the phosphorylation of IκB but not IRF3 was increased by MRP (Fig 1G and 1H). These data suggested that in Huh7 cells MITA activated the IFN-β signaling pathway through IRF3, while MRP activated the NF-κB pathway.

### MITA/STING and MRP inhibited HBV replication in Huh7 cells

**Fig 2:**
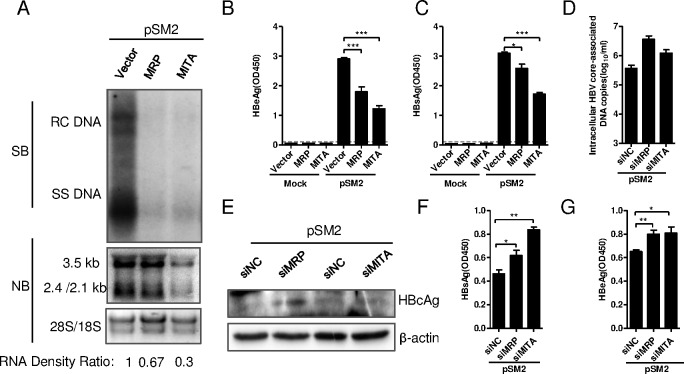
MITA/STING and MRP inhibited HBV replication *in vitro*. (A-C) The overexpression plasmid pFlag-MITA/STING or pHA-MRP was co-transfected with HBV plasmid pSM2 into Huh7 cells. (A) HBV DNA replicative intermediates (upper) and mRNAs (lower) were detected by Southern blot and Northern blot, respectively. HBV mRNA density signals on Northern blot were normalized to 18S/28S and showed as RNA density ratio. (B) Levels of HBeAg and (C) HBsAg secreted into supernatant were detected by ELISA after 5-fold dilution. Results were presented as the optical density at 450 nm (OD450). (D-G) siRNA specific to human MITA/STING or MRP was transfected into Huh7 for twice. pSM2 was cotransfected with siRNA at the second time. (D) Intracellular HBV core-associated DNA was extracted and quantified with real-time PCR. (E) Intracellular HBV core protein was detected by Western blot. (F) HBsAg and (G) HBeAg expressed in supernatant were diluted 5-fold and measured by ELISA. The dashed line represents the cutoff value (CoV), which was assumed to be 2.1-fold mean value of the negative samples. Significant differences were analyzed using a two-tailed unpaired *t*-test (*, P <0.05; **, P <0.01; ***, P <0.001).

**Fig 3:**
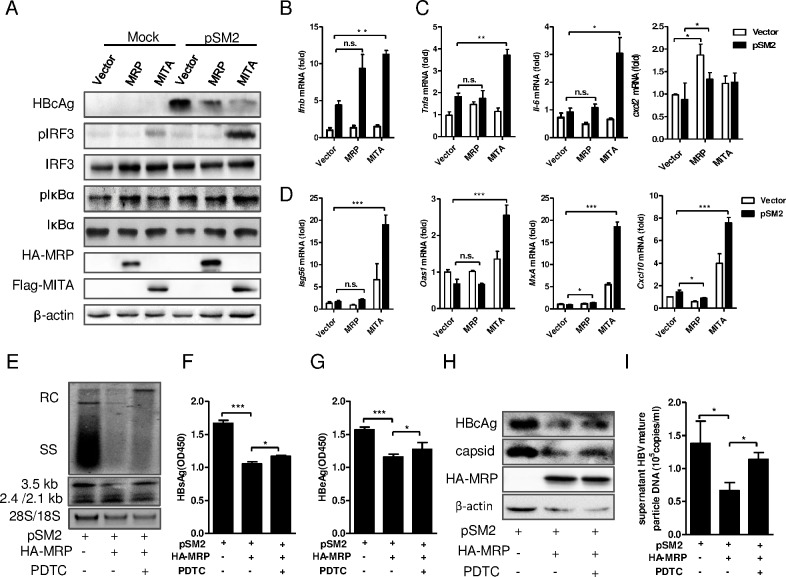
MITA/STING and MRP suppressed HBV replication by inducing innate immune signaling. (A-D) The overexpression plasmid pFlag-MITA/STING or pHA-MRP was co-transfected with HBV plasmid pSM2 into Huh7 cells. (A) Intracellular HBV core protein, pIRF3, IRF3, pIκB and IκB protein levels were detected by Western Blot with indicated antibody. The expression of MRP and MITA/STING were detected with anti-HA and anti-Flag antibody, respectively. (B) The levels of *Ifnb*, (C) pro-inflammatory cytokines *Tnfa, Il-6*, chemokine *Cxcl2* and (D) ISGs *Isg56*, *Oas1*, *MxA*, *Cxcl10* mRNAs in MRP or MITA overexpressed with or without pSM2 transfected Huh7 cells were detected by qRT-PCR. (E-I) HepG2 cells were transfected with pHA-MRP and pSM2 together and then treated with PDTC. (E) HBV DNA replicative intermediates and mRNA transcripts were detected by Southern blot and Northern blot, respectively. (F) The HBsAg and (G) HBeAg level in supernatant were measured by ELISA after 5-fold dilution of the supernatant. (H) HBcAg and capsid were detected by Western blot. (I) The supernatant mature virions were immunoprecipitated with anti-HBs antibodies and subjected to HBV core-associated DNA extraction, then quantified by real-time PCR. Significant differences were analyzed using a two-tailed unpaired *t*-test (*, P <0.05; **, P <0.01; ***, P <0.001).

To investigate the potential roles of MITA/STING and MRP in HBV replication, the HBV replication plasmid pSM2 was cotransfected with pFlag-MITA or pHA-MRP into Huh7 cells [43]. HBV replication was analyzed by monitoring HBV replicative intermediates, transcripts and the expression levels of HBeAg, HBsAg and HBcAg. As shown in Fig 2A, HBV DNA replicative intermediates were significantly inhibited by both MITA/STING and MRP as detected by Southern blot (Fig 2A, upper panel). MITA/STING and MRP also inhibited the transcription of HBV mRNAs (Fig 2A, lower panel), the expression of HBeAg and HBsAg in supernatant (Fig 2B and 2C) as well as the expression of the intracellular HBV core protein (Fig 3A). In all cases, MITA/STING expression achieved a much stronger inhibition of HBV replication and gene expression than MRP.

To further confirm the inhibitory function of MITA/STING and MRP on HBV replication, siRNAs specific to human MITA/STING and MRP were transfected into Huh7 cells. Since the expression levels of MITA/STING and MRP in Huh7 cells are very low, the siRNAs were transfected twice at concentration of 80 nM to achieve the knockdown effect (data not shown). Silencing of MITA/STING and MRP increased the cytoplasmic HBV core-associated DNA levels as detected by real-time PCR (Fig 2D) and the amounts of intracellular HBc protein (Fig 2E). The levels of HBsAg and HBeAg in the supernatant were significantly elevated after MITA/STING and MRP silencing as well (Fig 2F and 2G). Altogether, these results indicated that both MITA/STING and MRP inhibited HBV replication in hepatoma cells. Clearly, however, MITA overexpression resulted in a more significant inhibition of HBV replication than MRP overexpression did.

### MITA/STING and MRP suppressed HBV replication by inducing innate immune signaling in Huh7 cells

The activation of innate immune signaling in Huh7 cells by MITA/STING and MRP was analyzed at the present of the HBV replication. It is notable that HBV replication did not significantly induce the IFN-β pathway as the phosphorylation level of IRF3 did not change after pSM2 transfection. However, co-transfection of pMITA and pSM2 induced greater phosphorylation of IRF3 compared with that induced by pMITA expression alone (Fig 3A). The phosphorylation level of IκB was slightly increased by pMRP, pMITA and pSM2 transfection (Fig 3A). The mRNA levels of IFN-β, pro-inflammatory cytokines TNF-α and IL-6, and several ISGs, such as ISG56, OAS1, MxA and CXCL10, were significantly increased in MITA/STING and pSM2 co-transfected cells (Fig 3B–3D), suggesting that both NF-κB and IFN-β were activated when MITA/STING and pSM2 were co-expressed. In contrast, the IFN-β or ISGs mRNA levels were not changed in pMRP and pSM2 co-transfected Huh7 cells (Fig 3B and 3C), while the chemokine CXCL2 mRNA but not TNF-α or IL-6 was significantly up-regulated (Fig 3C), implying that MRP inhibited HBV replication via a mechanism that was different from MITA/STING and independent of IFN induction.

To further confirm the role of NF-κB activation in the anti-HBV effect of MRP, the inhibitor PDTC was used to block NF-κB activation. As shown in Fig 3E, the HBV DNA replicative intermediates and the HBV mRNA transcripts, which were inhibited by MRP, were partially rescued by PDTC treatment. PDTC also partially restored the HBsAg, HBeAg levels in supernatant (Fig 3F and 3G) and the intracellular HBcAg level (Fig 3H upper). Interestingly, the HBV capsid formation as well as the matured HBV particle level in supernatant (Fig 3H lower panel and 3I) was significantly rescued by PDTC. These results demonstrated that the activation of NF-κB by MRP was important for the anti-HBV effect of MRP.

### MITA/STING, MRP and c-di-GMP inhibited HBV replication in HepG2.2.15 cells

**Fig 4:**
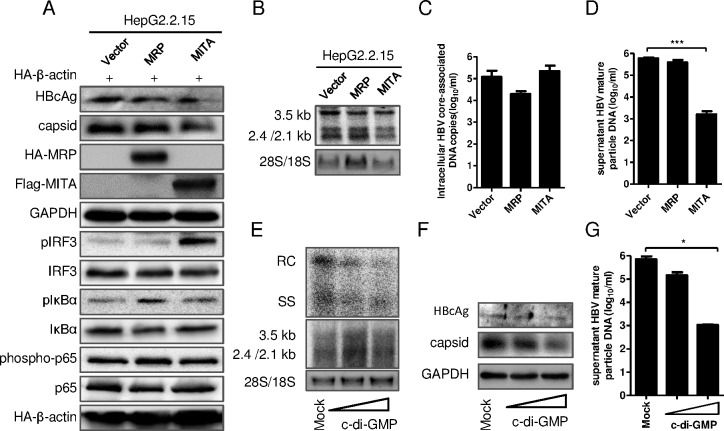
MITA/STING, MRP and c-di-GMP inhibited HBV replication in HepG2.2.15 cells. (A-E) The overexpression plasmid pHA-MRP, pFlag-MITA/STING or vector plasmid was transfected into HepG2.2.15 cells. The pHA-β-actin was cotransfected as an inner control. (A) The levels of HBV capsid, intracellular core antigen, pIRF3, IRF3, pIκBα, IκBα, phospho-p65, p65, MRP, MITA, GAPDH and β-actin were detected by immunoblotting with indicated antibodies. (B) The HBV mRNA replicative intermediates were detected by Northern blot. (C) Intracellular HBV core-associated DNA were extracted and quantified with real-time PCR. (D) The HBV mature particles in supernatant were immunoprecipitated with anti-HBs, and then HBV core-associated DNA were extracted and quantified with real-time PCR. (E-G) HepG2.2.15 cells were transfected with c-di-GMP of different concentrations and incubated for 24 h. (E) The HBV DNA replicative intermediates (upper) and mRNAs (lower) were investigated by Southern blot and Northern blot, respectively. (F) HBcAg (upper) and HBV capsid (lower) were immunoblotted with indicated antibody. (G) The supernatant HBV mature particles were immunoprecipitated and HBV core-associated DNA were extracted and quantified with real-time PCR. Significant differences were analyzed using a two-tailed unpaired *t*-test (*, P <0.05; **, P <0.01; ***, P <0.001).

The effects of MITA/STING and MRP on HBV replication were further studied in HepG2.2.15 cells. The overexpression of MITA/STING enhanced the phosphorylation level of IRF3, while MRP enhanced the phosphorylation level of IκB and p65, suggesting that the activation of innate immune responses in HepG2.2.15 cells by MITA/STING and MRP were different (Fig 4A). The intracellular HBcAg, HBV capsid level (Fig 4A) and the transcription of HBV mRNAs (Fig 4B) were reduced by MITA/STING and MRP overexpression. Consistent with the reduced capsid level, the intracellular HBV core-associated DNA level was decreased in MITA/STING and MRP overexpressing cells (Fig 4C). Interestingly, the mature particles in supernatant were strongly inhibited by MITA/STING but not MRP overexpression (Fig 4D). It’s clear that both MITA/STING and MRP have an inhibitory effect on HBV replication in HepG2.2.15 cells, while MITA/STING showed a stronger effect than MRP, consistent with the results in pSM2 transfected Huh7 cells.

To further confirm the control of HBV replication by MITA/STING pathway, the HBV replication was analyzed in HepG2.2.15 cells treated with c-di-GMP, the ligand of MITA/STING. The HBcAg protein expression, capsid formation, HBV DNA replicative intermediates in cells and the mature particles in supernatant were decreased by c-di-GMP treatment (Fig 4E–4G), although the HBV mRNA transcript level in cells was not apparently changed by c-di-GMP treatment (Fig 4F lower). These results demonstrated that c-di-GMP treatment inhibited HBV replication, which was in line with that MITA/STING overexpression behaved.

### MITA/STING and MRP suppressed HBV replication in mice

**Fig 5:**
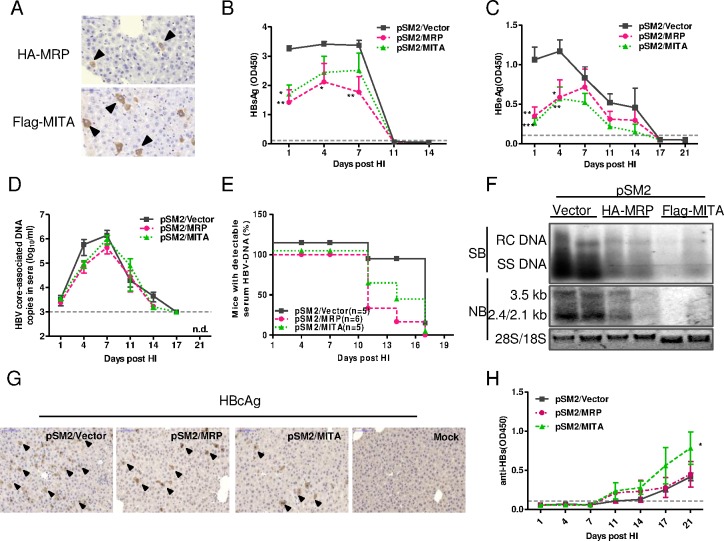
Overexpressed MRP and MITA/STING significantly suppressed HBV replication *in vivo*. 6~8 weeks old wild type C57BL/6 mice were hydrodynamically co-injected with 10 μg pSM2 plasmid and 10 μg pHA-MRP or pFlag-MITA/STING or control plasmid by tail-vein. (A) Expression of MRP or MITA/STING protein was detected in liver sample collected at 4 days post-HI by IHC staining with antibody against HA- or Flag-tag. (B) The levels of HBsAg or (C) HBeAg in 10-fold diluted sera were detected by ELISA. (D) The serum HBV DNA in mice was quantified by real-time PCR at the indicated time points. The detection limit for HBV DNA in our system was 1000 copies per milliliter. (E) The percentage of mice with detectable serum HBV DNA at indicated time point was calculated as the percentage of total mice in each group. (F) HBV DNA and RNA replicative intermediates in liver samples collected at 4 days post-HI were measured with Southern blot and Northern blot, respectively. Each lane represented a liver sample from a mouse. (G) Immunohistochemical staining showed HBcAg levels in liver at 4 days post-HI. (H) The dynamic levels of antibody specific to HBV S antigen in 10-fold diluted sera at indicated time points were detected by ELISA. The black arrow indicated the HA-MRP, Flag-MITA or HBcAg expression positive hepatocytes. The dashed line represents the cutoff value, which was assumed to be 2.1-fold mean value of the negative samples. Five or six mice per group were analyzed. The two ways ANOVA followed by Bonferroni’s test was used to determine the differences in multiple comparisons (*, P <0.05; **, P <0.01; ***, P <0.001).

We further investigated the influence of MRP and MITA/STING on HBV replication using a hydrodynamic injection mouse model. Empty vector pcDNA 3.1(+), pMRP or pMITA was mixed with pSM2 and were delivered into wild type C57BL/6 mice by HI. The expression of MRP and MITA/STING was then detected by immunohistochemical staining (IHC) with anti-HA and anti-Flag antibodies, respectively (Fig 5A). After HI of plasmid mixtures into C57BL/6 mice, serum HBsAg levels were monitored from day 1 through day 21 post-HI. A high serum HBsAg level was detected in mice receiving pSM2/Vector at 1 through 7 days post-HI (Fig 5B). Although seroconversion occurred at 11 days post-HI in all treatment groups, serum HBsAg levels as well as HBeAg levels, were significantly lower in mice that received pSM2/pMITA and pSM2/pMRP than in mice that received pSM2/Vector (Fig 5B and 5C). The levels of serum HBV core-associated DNA reached a peak at day 7 post-HI and were undetectable at day 17 post-HI in all treatments. A reduction in serum HBV core-associated DNA was observed in pMITA- and pMRP-delivered mice at day 4 post-HI (Fig 5D). Compared with control group, the percentages of mice with detectable serum HBV DNA were reduced in the pSM2/MITA and pSM2/MRP groups at day 10 and day 14 post-HI (Fig 5E).

The intrahepatic HBV DNA replicative intermediates and mRNAs at day 4 post-HI were detected by Southern blot and Northern blot, respectively. The results showed an obvious reduction of HBV DNA and RNA levels in the pSM2/MITA and pSM2/MRP groups (Fig 5F). Furthermore, the percentages of HBcAg-positive hepatocytes in liver tissue were also decreased significantly in the pSM2/pMITA and pSM2/pMRP groups compared with the pSM2/Vector group as shown by IHC staining (Fig 5G). Interestingly, the level of antibodies specific for HBsAg (anti-HBs) in serum was increased in the pSM2/pMITA group, suggesting that MITA/STING enhanced HBV-specific antibody production *in vivo* (Fig 5H). These data demonstrated that both MITA/STING and MRP inhibited HBV replication, and that MITA/STING played a role in triggering HBV-specific adaptive immune responses.

### HBV replication was enhanced in MITA/STING knockout mice

**Fig 6:**
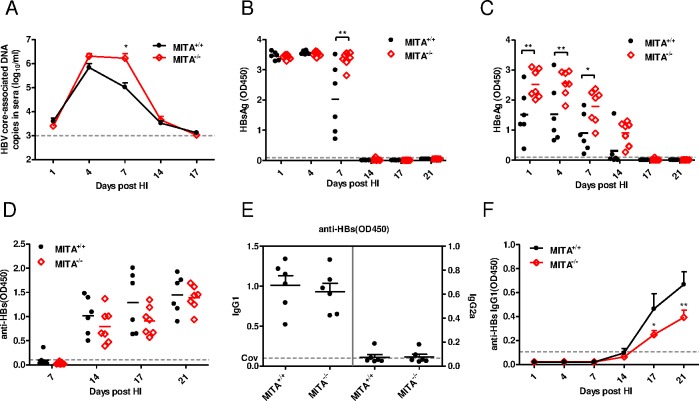
MITA deficiency induced elevation of serum HBV viral loads and impaired HBV humoral immune response *in vivo*. Wild type (MITA^+/+^) and MITA knockout (MITA^-/-^) mice were hydrodynamically (HI) injected with 10 μg pSM2 plasmid. (A) The serum HBV DNA in mice was quantified by real-time PCR at the indicated time points. (B) The levels of HBsAg and (C) HBeAg in 10-fold diluted sera were detected by ELISA. (D) The dynamic anti-HBs antibodies in 10-fold diluted sera were monitored and detected by ELISA. (E) The levels of HBsAg-specific IgG subtypes were determined by ELISA. (F) The dynamic of HBsAg-specific IgG1 subtype were also detected by ELISA. The dashed line represents the cutoff value, which was assumed to be 2.1-fold mean value of the negative samples. Six or seven mice per group were analyzed. The two ways ANOVA followed by Bonferroni’s test was used to determine the differences in multiple comparisons (*, P <0.05; **, P <0.01).

The influences of MITA/STING and MRP on HBV replication were further analyzed in MITA/STING^-/-^ and wild type littermate mice (MITA/STING^+/+^). The HBV replication plasmids were delivered into mice by HI. The quantification of HBV core-associated DNA copies in mouse serum samples showed that HBV DNA levels were higher in MITA/STING^-/-^ mice than in wild type mice at day 7 post-HI (Fig 6A). The ELISA results showed that the HBsAg level in serum of MITA/STING^-/-^ mice was also higher at day 7 post-HI (Fig 6B). Compared with that in wild type mice, the serum HBeAg level in MITA/STING^-/-^ mice was higher during all infection courses (Fig 6C), indicating an enhanced replication of HBV in MITA/STING^-/-^ mice.

### HBV antibody response and specific T-cell immune response in MITA/STING knockout mice

MITA/STING as an adapter protein not only instigates transcription of cytoplasmic DNA-induced cellular defense genes but also facilitates adaptive responses required for viral control and elimination [44]. Therefore, we assessed the production of anti-HBs antibodies in MITA/STING^+/+^ and MITA/STING^-/-^ mice receiving pSM2. As shown in Fig 6D, following the decline of serum HBsAg, the anti-HBs levels rose after day 7 post-HI and increased thereafter in both groups. However, the antibody level in MITA/STING^-/-^ mice was weaker compared to that in MITA/STING^+/+^ mice, which was consistent with previous findings that overexpression of MITA/STING enhanced HBV-specific antibody levels in wild type mice (Fig 5H). IgG1/IgG2a antibody responses were measured as an indicator of the Th bias of specific immune responses [45]. The different subtypes of HBsAg-specific IgGs present post-HI indicated that pSM2 induced a significantly stronger Th2 response (Fig 6E). Then, the HBsAg-specific IgG1 subtype was monitored at different time points after HI, and results showed that the IgG1 response was weakened in MITA/STING^-/-^ mice (Fig 6F).

**Fig 7:**
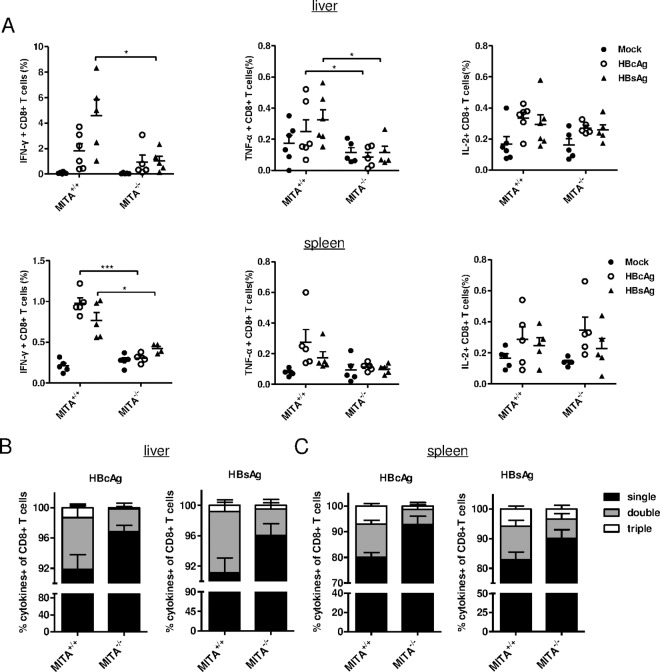
Liver-infiltrating CD8^+^ T lymphocytes and splenocytes in MITA knockout mice displayed deficient cytokines producing phenotype and impaired specific CTL response. (A) Wild type (MITA^+/+^) and MITA knockout (MITA^-/-^) mice were hydrodynamically injected with pSM2 plasmids. Twenty-eight days after injection, intrahepatic lymphocytes and splenocytes from HBsAg-positive mice were isolated and unstimulated (Mock) or stimulated with HBcAg- or HBsAg-derived peptides *ex vivo*. The frequencies of IFN-γ+ or TNF-α+ or IL-2+ CD8+ T cells were measured by intracellular staining and analyzed by FACS assay. (B) Quantitative analysis showed the percentages of one or two or three kinds of cytokines (IFN-γ/ TNF-α/ IL-2) simultaneously producing cells within the CD8+ T cell population from liver or (C) spleen. Five or six mice per group were analyzed. Significant differences were analyzed using a two-tailed unpaired *t*-test (*, P <0.05; **, P <0.01; ***, P <0.001).

We also analyzed HBV-specific CD8^+^ T cell response. Intrahepatic lymphocytes and splenocytes were isolated at day 28 post-HI, and the cells were stimulated with H-2k^b^-restricted CTL epitope peptides derived from HBsAg or HBcAg. The populations of HBsAg- or HBcAg-specific IFN-γ-, TNF-α-, and IL-2-producing CD8^+^ T cells were measured by intracellular cytokine staining and analyzed via flow cytometry. Compared to those in MITA/STING^+/+^ mice, the percentages of HBcAg- and HBsAg-specific mono-functional CD8^+^ T cells producing IFN-γ or TNF-α among the liver-infiltrating CD8^+^ T cells were reduced in MITA/STING^-/-^ mice. In addition, the frequency of IFN-γ^+^ CD8^+^ T cells was also distinctly lower among spleen CD8^+^ T cells of MITA/STING^-/-^ mice than that of MITA/STING^+/+^ mice. There was little difference in the levels of IL-2-producing CD8^+^ T cells between the two groups in the liver as well as in the spleen (Fig 7A). The majority of HBV peptide-stimulated CD8^+^ T cells in the MITA/STING^+/+^ and MITA/STING^-/-^ mice were monofunctional, producing one cytokine (IFN-γ, TNF-α or IL-2). However, the percentages of polyfunctional CD8^+^ T cells, which simultaneously produced two or three cytokines (IFN-γ/ TNF-α/ IL-2), were significantly reduced among the liver-infiltrating CD8^+^ T cells, as well as among the spleen CD8^+^ T cells isolated from the MITA/STING^-/-^ mice compared with that observed from the MITA/STING^+/+^ mice (Fig 7B and 7C). These findings suggest that the cytokine producing function of CD8^+^ T cells was impaired when MITA/STING was deficient. Taken together, a MITA/STING deficiency impaired HBV-specific antibody responses and reduced the intensity of the CD8^+^ T cell response.

## Discussion

Previously, we found that despite a negative modulation of MITA/STING induced IFN-β activation, MRP played a different role in response to RNA and DNA virus infection since MRP blocked MITA-mediated IFN signaling pathway induced by SeV infection but enhanced HSV-1 induced IFN response [29]. In this present study, we found that MITA/STING and MRP overexpression inhibited HBV replication *in vitro* and *in vivo*. In addition to activating the innate immune response, MITA/STING also regulates the adaptive immune response to HBV.

Our study found that MITA/STING was a better inhibitor of HBV replication than MRP (Fig 2 and Fig 4), which could be attributed to the different signaling pathways and cytokine profiles activated by MITA/STING versus MRP. While MITA/STING was able to activate the IFN-β pathway through IRF3 phosphorylation and consequently increase the expression of ISGs, pro-inflammatory cytokines and chemokines, MRP was able to active the NF-κB reporter, up-regulate IκB phosphorylation and chemockine CXCL2 expression in Huh7 cells. Although MITA/STING overexpression did not activate the NF-κB reporter in Huh7 cells, it stimulated the IFN-β promoter whose activation requires the cooperative action of IRF3 and NF-κB. It may possibly attribute to that the baseline of NF-κB activation in Huh7 cells is higher than in other cell lines because p65 phosphorylation was readily detectable in Huh7 cells without stimulation. This phenomenon is consistent with previous reports that NF-κB is constitutively active in hepatoma tissues and Huh7 cells [46]. Activations of both IFN-β and NF-κB signaling have been shown to inhibit HBV replication through the induction of different effectors. A number of previous studies demonstrated that type I IFN could induce ISGs, such as MxA and IFIT1/2 (ISG56/ISG54), and suppress HBV replication *in vitro* and *in vivo* [47–49]. Generally, activation of the NF-κB pathway leads to the production of pro-inflammatory cytokines, such as TNF-α, which has a known direct anti-viral effect on HBV. TNF-α was shown to suppress HBV replication by damaging the formation or stability of cytoplasmic viral capsids [50]. In the present study, MRP reduced the levels of HBV DNA replicative intermediates strongly and the levels of HBV transcripts slightly. In contrast, MITA/STING significantly suppressed the formation of both HBV DNA replicative intermediates and transcripts. The precise mechanism of how MITA/STING and MRP function in this capacity needs further investigation.

The presence of HBV significantly enhanced the MITA/STING-mediated IRF3-IFN pathway, as shown by the increased level of phosphorylated IRF3, the upregulation of IFN-β mRNA and the elevated expression levels of the downstream ISGs, pro-inflammatory cytokines and chemokines (Fig 3). These findings suggest that the MITA/STING signaling pathway may sense HBV components and in turn be further activated. Indeed, Dansako *et al*. demonstrated that HBV-derived synthetic dsDNA and the HBV virus could induce ISG56 expression in a cGAS/STING-dependent manner [51]. Consistently, Cui *et al*. showed that HBV DNA could be detected by DNA sensors, specifically the cGAS/STING signaling pathway, in an immortalized mouse hepatocyte cell line [19]. On the other hand, HBV was shown to counteract the MITA/STING-mediated antiviral responses by disrupting K63-linked ubiquitination of MITA/STING [24].

The innate immunity signals are essential for the development of adaptive immune response [52]. Soon after discovery, MITA/STING was found to be required for efficient T-cell response to antigen induced by DNA-vaccine and CTL response triggered by baculovirus [26, 53]. A recent report also showed that activation of the cGAS-STING pathway boosts antigen-specific T cell activation and antibody production in mice [54]. Consistent with previous reports, our results showed that MITA/STING deficiency impaired HBV-specific humoral and T-cell mediated responses in HI mouse model, indicated by the weakened production of HBV-specific antibodies and the multiple cytokines producing ability of CD8^+^ T cells specific to HBV in MITA/STING knockout mice. On the other hand, overexpression of MITA/STING enhanced the antibody response to HBV *in vivo*.

In the HI mouse model, overexpression of MITA/STING and MRP restricted HBV replication and the deficiency of MITA/STING enhanced HBV replication. Thomsen *et al*. also showed that overexpression of MITA/STING in mice hepatocytes could reduce HBV replication. However, they found that MITA/STING deficiency has no influence on HBV replication in a adenovirus-HBV model [55]. The discrepancy may attribute to the different mechanisms of host recognition and responses to systemic infected adenovirus vector and naked plasmid DNA delivered by HI. Adenovirus DNA was demonstrated to induce IFN-β production by TLR9/MyD88 cascade in plasmacytoid dendritic cells (pDCs) and MyD88-independent pathway in conventional DCs and macrophages [56]. Daniela *et al*. demonstrated that cGAS/STING pathway was essential in MyD88-independent pathway for type I IFN induction and antiviral response to recombinant adenovirus (rAdV). Although the rAdV could trigger robust type I IFN production through STING-IRF3 pathway as well as Ad-HBV proved by Thomsen [55], loss of cGAS/STING minimally impacted viral clearance and persistence of transgene expression delivered by rAdV [57], which indicated that cGAS/STING was dispensable to the induction of adaptive immune response to rAdV. The minimal impact of MTIA/STING-mediated type I IFN pathway on adaptive immune response to rAdV was also previously confirmed in STAT2 knockout Syrian hamster model and IFNRI and STING knock out mouse [58, 59]. Different from the receptor mediated uptake of rAdV DNA, the naked HBV plasmids were delivered directly into cytoplasm by hydrodynamic injection. In the cytoplasm, the HBV DNAs were mainly recognized by cGAS/STING firstly, especially in MITA/STING-abundant nonparenchymal cells (NPCs). Since hepatocytes express low level of MITA/STING, the enhanced HBV replication in MITA/STING^-/-^ mice probably was due to the deficiency of MITA/STING in NPCs, as lack of MITA/STING would influence host innate immune response as well as DCs maturation, and defective antigen presentation of DCs consequently impaired the expansion of leukemia specific T cells and CTL response to control HBV replication.

In conclusion, although the baseline of MITA/STING expression was low in hepatoma cells, the complement of MITA/STING by overexpression restricted HBV replication significantly, suggesting an important role of MITA/STING in HBV persistence. The spliced variant of MITA/STING, MRP also inhibits HBV replication *in vitro* and *in vivo*, via the activation of NF-κB pathway. Deficiency of MITA/STING weakened HBV specific humoral and CTL responses, and consequently enhanced HBV replication in MITA/STING^-/-^ mice, which indicated indispensable role of MITA/STING in adaptive response initiation. Due to the important role of MITA/STING in linking the innate response to adaptive response, the agonists to MITA/STING have been explored as vaccine adjuvants and for tumor treatment. Similarly, the MITA/STING agonists probably could be used as adjuvants of therapeutic vaccination for chronic HBV infection.
